# Decision aids for respite service choices by carers of people with dementia: development and pilot RCT

**DOI:** 10.1186/1472-6947-12-21

**Published:** 2012-03-19

**Authors:** Christine Stirling, Susan Leggett, Barbara Lloyd, Jenn Scott, Leigh Blizzard, Stephen Quinn, Andrew Robinson

**Affiliations:** 1 Wicking Dementia Research and Education Centre, Menzies Research Institute, University of Tasmania, Private Bag 121, Hobart, TAS, Australia 7000; 2 School of Nursing and Midwifery, University of Tasmania, 24 Campbell St, Tasmania, Australia; 3 School of Psychology, University of Tasmania, Sandy Bay Campus, Tasmania, Australia; 4 Menzies Research Institute, University of Tasmania, 17 Liverpool St, Hobart, Australia; 5 Flinders Clinical Effectiveness, Flinders University, Adelaide, Australia

**Keywords:** Decisional conflict, Decision aid, Dementia, Carer, Community services, Randomised-control trial, Qualitative

## Abstract

**Background:**

Decision aids are often used to assist individuals confronted with a diagnosis of a serious illness to make decisions about treatment options. However, they are rarely utilised to help those with chronic or age related conditions to make decisions about care services. Decision aids should also be useful for carers of people with decreased decisional capacity. These carers' choices must balance health outcomes for themselves and for salient others with relational and value-based concerns, while relying on information from health professionals. This paper reports on a study that both developed and pilot tested a decision aid aimed at assisting carers to make evaluative judgements of community services, particularly respite care.

**Methods:**

A mixed method sequential study, involving qualitative development and a pilot randomised controlled trial, was conducted in Tasmania, Australia. We undertook 13 semi-structured interviews and three focus groups to inform the development of the decision aid. For the randomised control trial we randomly assigned 31 carers of people with dementia to either receive the service decision aid at the start or end of the study. The primary outcome was measured by comparing the difference in carer burden between the two groups three months after the intervention group received the decision aid. Pilot data was collected from carers using interviewer-administered questionnaires at the commencement of the project, two weeks and 12 weeks later.

**Results:**

The qualitative data strongly suggest that the intervention provides carers with needed decision support. Most carers felt that the decision aid was useful. The trial data demonstrated that, using the mean change between baseline and three month follow-up, the intervention group had less increase in burden, a decrease in decisional conflict and increased knowledge compared to control group participants.

**Conclusions:**

While these results must be interpreted with caution due to the small sample size, all intervention results trend in a direction that is beneficial for carers and their decisional ability. Mixed method data suggest the decision aid provides decisional support that carers do not otherwise receive. Decision aids may prove useful in a community health services context.

**Trial registration number:**

ISRCTN: ISRCTN32163031

## Background

Decision Aids (DAs) are intended to help individuals participate in or make health care choices in situations where outcomes may be indeterminate or dependent on values and beliefs [1,2]. Effective DAs have been shown to improve knowledge, reduce indecision, increase decision making involvement and lower decisional conflict [1]. Over 500 aids have been developed globally [1], but these have largely focused on 'rational' screening or treatment choices for conditions such as cancer and heart disease. DAs are rarely used to help those with chronic or age related conditions to make decisions about care services, and yet they represent a potentially useful resource for care-related choices in complex chronic disease situations.

Dementia is a progressive illness, resulting among other outcomes in decreasing decisional capacity. As many as 26% of people with dementia (PWD) remain living in the community, the vast majority cared for by one or more family members [3] who increasingly take responsibility for care decisions. The experience of caregiving, which is likely to include assistance with communication, cognition and emotion, as well as with mobility, self-care and other activities of daily living [3], has been described as an "unremitting burden" [4]. This burden is correlated with poor health outcomes, such as depression and anxiety and an overall increased risk of morbidity and mortality for carers, and earlier institutionalisation for care-recipients [5-8].

Services such as respite, either in the PWD's home, in a day care centre or in a residential facility, can reduce this burden, easing-albeit temporarily-carers' physical and emotional workload [9-16]. However, respite services are under used, with only 30% of carers of PWD reporting having used a respite service even where referral has been made and services are readily accessible [3,17-19]. Carer service choices are affected by a complex array of other factors. These include individual personality traits - carers, for example, have varying levels of tolerance for their situations [20] and the emotional, physical and financial costs of caring [20,21].

A number of authors argue that carers need to give themselves 'permission' to use respite, trusting that the person they look after is being provided with quality care [19,22-26] and that their own burden is real and in need of alleviation. In addition, Toseland et al. [27] suggest that health care workers can help reduce some of the barriers to accepting assistance by developing trust, adequate collaborative planning, providing information and advice, listening, clarifying for caregivers the nature of respite, and providing emotional support. At the same time, carers may gain a greater sense of control [28] if they are given the opportunity to "determine what respite means for them, and which services can best meet their needs" [29], p. 303.

Carers need access to realistic, contextually relevant information in order to make service-related decisions, and may need support to weigh up available options. Carers of PWD are therefore a suitable group for targeted DAs, given that they experience many conditions that are known to cause emotional turmoil and consequent delayed decision making [30,31]. DAs typically contain pertinent information about the condition or disease, both the advantages and disadvantages of treatment options, activities such as weigh scales that help individuals to clarify their values by asking them to rate their perceptions of the benefits and disadvantages of different treatment or service options, and advice about the decision-making process [32]. DAs constitute appropriate resources in the dementia carer's situation and could play a central role in helping carers to make decisions about respite services.

This two stage study developed and trialled a respite service decision aid to assist carers of PWD when making judgements about community services. The decision aid, called the GOLD Book (Guiding Options for Living with Dementia), targets primary carers of community dwelling PWD.

### Ethics

The two stage research was approved by the Tasmanian Human Research Ethics Committee. All study participants received an information sheet explaining the nature of the project, as well as benefits and risks of participation, and completed a consent form prior to the first round of data gathering.

## Stage 1: Methods 1

We adopted a qualitative research design for the development of the DA, and followed the process recommended in the Ottawa guidelines [32]. The key steps in this process are: identify need; assess feasibility; identify the objectives of the DA; identify the framework and methods of decision support to be used; and select suitable evaluation measures. Data collection involved a review of the literature, consultations with an expert advisory panel, semi-structured interviews, and focus groups, which informed the development of the decision aid. Sample size and formats recommended in the Ottawa guidelines [32] were followed. The literature review identified relevant research on the topic of dementia community services, respite services, care outcomes, and carer stress. The expert panel of community service providers included key figures from three national non-government organisations in the field of carers and dementia, one of which focused on the disease itself, one on carers and one on respite services. These advisors provided access to 'expert' but non-academic voices on dementia support services for carers. Expert panel participants were asked to review the DA and to contribute their perspectives during qualitative interviews.

A convenience sample of 13 experienced carers was sourced to provide insights into the perspectives of the primary intended users of the DA. Fifteen carers who had participated in previous unconnected studies conducted by the Wicking Centre, and had indicated their willingness to be contacted again, were invited to participate. Thirteen of these agreed to participate. Our carer participants had characteristics consistent with the demographic profile of carers of PWD in Australia [3,33]. Most Australian data collections about co-resident carers of PWD are small and unrepresentative, but data collation shows that about 70% will be female, with 95% being older than 60 years of age, and that 65% will be spouses of the PWD and 30% a relative [3]. In our sample, females and spouses were over-represented as most carers were female (85%), the spouse of the care recipient (85%) and aged over 66 (77%).

Face-to-face interviews took between one and two hours and were conducted by one of the authors (BL). Questions focused on eliciting carers' perceptions of the usefulness, content and style of the DA and of their own information and decision making needs. Three focus groups were held with three groups of health care workers (publicly funded community nurses (n = 4), non-government organisation counsellors (n = 4), and privately funded home visiting support workers (n = 4) from community health service providers. The aim of the focus groups was to understand if and how those professionals most likely to have access to the DA might use the aid, with a view to evaluating which components might improve uptake of the intervention. Groups were chosen to be homogenous in terms of occupation and were recruited through advertising with their employing institutions. The ensuing 12 voluntary participants were mostly female (11 females: 1 male) and had an average age of 50. The number of actual participants per group (4) was a coincidental result, as we had aimed to recruit 6-8 participants per group. Authors CS and BL facilitated the focus groups using a question guide. The qualitative data was audiotaped and notes were extracted from these tapes by BL after consultation and methodological consensus with CS. (Different aspects of this and other data have been reported in Lloyd and Stirling [34]). Specific feedback was incorporated into the final draft of the DA in an iterative process. This involved creating two further drafts and presenting them to the project Steering Committee and three experts for further feedback.

## Stage 1: Results 1

A workbook format was selected for the GOLD Book DA as it was anticipated that paper based approaches would be more accessible for our target group of older community dwelling carers. The DA has a typical structure and contains brief descriptive information about the common community services available (such as domestic help, gardening and maintenance, personal care), descriptive information about respite care, decision tools based on selecting a respite care option, vignettes describing carers' experiences, brief targeted information about the trajectory of decline in dementia, and phone numbers and links to facilitate gaining further information. Step by step 'weigh scales' (adapted from [35] with permission) that allow users to weigh up service preferences by clarifying their own needs are included. The benefits and disadvantages of each respite service option are listed. In order to accommodate carers who may not want more information about dementia at the time of initial use, the 'knowledge about dementia' section is located at the back of the book and is clearly identified. These elements derived from cumulative information gained from the literature, expert advisors, health professionals and carers who participated in the research.

We encountered two key areas of difficulty in providing 'evidence based' information in the DA around dementia outcomes and service availability. Information about dementia outcomes is often ambiguous, due to a lack of research evidence about the impacts of causative factors, the diversity of behavioural manifestations, and uncertainty about decline rates and time to death [3]. This meant the decision aid could not link care options with firm probabilities, but relied instead on a known varied trajectory of decline and death [3,35] to provide important information for carers attempting to make decisions about service needs into the future. The incurable and progressive nature of dementia was highlighted in the DA, with a focus on the decreasing cognitive and functional capacities of those affected and the consequent and inevitable result of death. The inclusion of realistic information was, however, contested by certain health workers as the following quote by an expert advisor demonstrates: '*Take out the vignettes... They won't want to read them because they'll think "I have enough problems of my own"' *(EA3). Carers, by contrast, valued the realistic information as they had frequently found it difficult to access through other means. This carer points out: *'The biggest problem for carers is-where do you get the information?' *(Mrs J). As developers, we prioritized the viewpoints of carers-who would be the primary users of the DA as a resource-over those of other project participants, consequently opting to include information about survival times and dementia decline in the DA in order to assist informed judgments.

The variability of service availability in different locations provided further difficulty for the development of the DA. As this community nurse focus group participant said: '*My only problem [with the DA] is the availability of service. It's so varied in different areas. Emergency respite-there is no such thing' *(FGCN). In Australia, access to services can vary depending on carers' financial situations, geographic locations, and the particular health service providers with which carers interact. This carer experienced a cap on services: '*I had a range of services and they were very good, but towards the end when I really needed help I was told that my entitlement had run out and I couldn't get any more*' (Mrs V). These variations meant that different participants reported dissimilar experiences as to the availability of services during the DA development, and opinions varied amongst our informers as to which services carers could access. Such inconsistencies in service availability made it difficult to provide nationally relevant information within the DA about services. Accordingly, the DA needed to offer generic advice as it was targeting a national audience. The DA directed carers to four key support organisations with national toll free phone numbers. The aim was to ensure that carers have improved access to consistent and up to date information about service availability.

The qualitative findings collected during the development and trial of the DA strongly suggest that the intervention provides carers with needed decision support. Most carers felt that the DA was useful because, as one said, *'it makes you think through decisions well'*. Vignettes of the experiences of other carers enabled participants to *'relate to the stories and how respite benefits carers'*. As well, carers had a better understanding of *'*... *the decisions I will need to make and the choice I will be facing*', as well as '... *the track it [dementia] takes and what services will be available to support [me]*'. Another said *'I really feel every carer needs one, it gives you the things you need to know'*. A common theme was that use of the DA would be enhanced by having someone with expertise to discuss decisions with after using the DA. Expert opinion was thus valued by carer participants as a resource complementing their own agency, rather than as a 'gatekeeping' entity shaping and perhaps constraining that agency.

## Stage 2: Methods 2

The pilot, parallel group, randomised control trial of the DA intervention, conducted during 2010, allowed us to trial our recruitment, intervention and data collection methods and assess the potential benefits of the intervention. We hypothesised that the DA would lower primary dementia carers' burden by improving their ability to make community service decisions.

### Setting for the trial

The trial was conducted in Tasmania, Australia's southern-most and least populous state.

### Population

**Figure 1:**
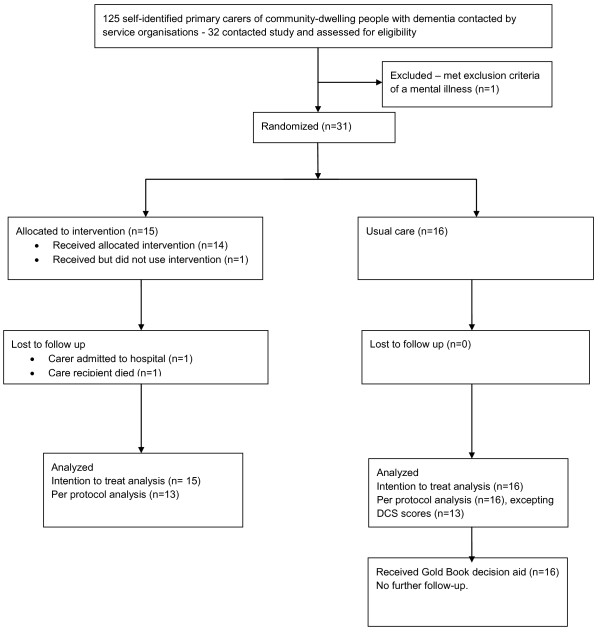
**Flow Diagram of GOLD Book pilot RCT**.

Eligible participants were self-identified primary carers of PWD, who were residing in the community and were free of any significant mental illness. Four providers of services to people with dementia and their carers were involved in the recruitment of study participants through developing a list of potential participants from their databases and sending a letter and project brochure to all known carers of PWD (n = 125). In addition, a small number of participants were recruited as a result of requests in the media for participants. We aimed to recruit 60 participants in order to fully trial the procedures for a main study, an noted that this happens to be similar sample sizes to other decision aid trials [2]. Thirty-one participants were recruited from April to August 2010 with the assistance of local service organisation mail-outs, with the lower than expected participation proportion of 25% failing to deliver the 60 participants initially targeted in the time permitted. Carers were excluded from the study if they reported (in response to a question early in the questionnaire) a psychiatric illness or medication use for a mood disorder (bi-polar, anxiety or depression). Those excluded at this point were provided with the decision aid at the end of the project. One respondent to the letter of invitation was excluded in this way (Figure 1).

Participants were randomly assigned following simple randomisation procedures (computer generated numbers) to either the intervention group (receive the DA in the mail, n = 15) or control group (DA received at the end of data collection, n = 16). Data were collected from carers via interviewer administered questionnaires. Baseline characteristics were similar across groups (See Table 1), with both having a mean age of 66.6 years, moderate to high levels of carer burden (mean 12.3/12.4 on a scale of 1-26), and mean decisional conflict of 20.5/19.5 on a scale of 0-100.

**Table 1:** Baseline Demographics of RCT Participants

	Decision Aid (n = 15) n (%)	Control Group (n = 16) n (%)
Gender		

male	4 (50.0)	4 (50.0)

female	11 (47.8)	12 (52.2)

Relationship with PWD		

spouse/life partner	8 (47.1)	9 (52.9)

child	7 (50.0)	7 (50.0)

Language other than English at home?		

yes	0 (0.0)	0 (0.0)

no	14 (51.9)	13 (48.1)

Age-mean years (range)	66.67 (44.00-90.00)	66.60 (42.00-85.00)

Length of time as a carer-mean years (range)	5.73 (1.00-20.00)	4.06 (1.00-9.00)

Highest level education		

Postgraduate	0 (0.0)	3 (18.8)

Degree	4 (26.7)	2 (12.5)

Diploma	0 (0.0)	3 (18.8)

Trade certificate	3 (20.0)	2 (12.5)

Year 11, 12, or equivalent	4 (26.7)	0 (0.0)

Secondary school	4 (26.7)	5 (31.3)

Primary school	0 (0.0)	1 (6.3)

Preferred Respite Option		

In-home respite	7 (46.7)	6 (37.5)

Day respite	3 (20.0)	4 (25.0)

Short-term residential respite	5 (33.3)	6 (37.5)

Decision Control Preference		

I make the decision	7 (46.7)	5 (31.3)

I make the final decision after seriously considering the opinion/s of other/s	5 (33.3)	8 (50.0)

I share responsibility for deciding	3 (20.0)	3 (18.8.0)

### Intervention

Following the baseline interview, intervention group participants were mailed the DA. Participants were given instructions asking them to work through the contents of the DA over the following week. Both groups continued to receive usual care from community services, and control group participants (wait-listed) were mailed a copy of the DA at the end of their data collection period, as receiving the information was considered potentially beneficial with no risk of harm; no further measures were taken after the control group received the DA. The initial interview was conducted face-to-face but subsequent data gathering was carried out over the telephone. While it was not possible to blind the researcher to participants' allocation in this study, the interviewer used interview sheets with standardised wording with pre-coded responses.

### Outcome measures

The effectiveness of the intervention was assessed by comparisons between control and intervention groups after three months participation in the trial. Data was collected at baseline, after two weeks (to assess the processes of using the DA), and three months post intervention (suitable time for changes to be evident) by interviewer administered questionnaires. The expected primary endpoint was a decrease in carer burden in the intervention group three months after receiving the DA. Secondary efficacy endpoints included reductions in decisional conflict, increased carer preference for involvement in decision making, and improved knowledge of dementia.

Information collected about the carer included demographics, relationship to the care recipient, carer age and education levels. Carer stress was measured through the Modified Carergiver Strain Index (MCSI), which is a short 13 item index first developed by Robinson [36] and modified by Thornton and Travis [37]. The MCSI examines both subjective and objective elements of caregiver burden with higher scores reflecting greater burden. The MCSI has good internal reliability (α - .90) and a test-retest reliability coefficient of .88 [37]. It is useful both for the identification of individual items that cause most difficulty and for a global score and has been used successfully with older caregivers of PWD [38].

Carer decisional conflict was measured through the Decisional Conflict Scale (DCS), which is a 16 item survey that yields a 5 scale measure of decisional uncertainty and factors contributing to uncertainty (feeling uninformed, being unclear about values, effectiveness of decision, and feeling unsupported in decision making) [39]. It has been used in more than 30 studies, discriminates between those who make or delay decisions, and has Cronbach's alpha coefficients exceeding 0.78 [39]. It is most useful in detecting change when a DA is compared to usual care as in this study [1].

Carer knowledge of the dementia trajectory was assessed through nine dementia knowledge questions developed specifically to address the knowledge content of the DA with potential responses of 'true', 'false' and 'don't know'.

Carer decision participation preferences were assessed through the Control Preference Scale [40]. Five response statements assess participants' preferred role in decision making, two responses represent an active role, one a shared role and two responses a passive role.

### Treatment fidelity

Intervention participants were asked if they had read and used the DA; only one participant had failed to use the DA, stating she was too busy to read it.

### Data analysis

All analyses of primary and secondary endpoint variables were carried out according to the randomised treatment arm (intention-to-treat) using last observation carried forward in place of missing data for two intervention group participants and three control group participants (Table 2), but with a complete case analysis provided for comparison (Table 3). Descriptive statistics of baseline characteristics of all caregivers were reported by randomised arm as mean and standard deviation for continuous variables, and as counts and percentages for categorical variables. The method of analysis used unpaired t-tests comparing mean changes in scaled measurements of carer stress (primary endpoint) and decisional ability and dementia knowledge (secondary endpoints) between baseline (Time 1) and three months post-intervention (Time 3) for caregivers in the two arms. All statistical tests were performed two-sided, and confidence intervals were calculated to provide 95% coverage. The statistician was not blinded to the allocation.

**Table 2:** Intervention results - Intention to Treat analysis (n = 31)

	Mean (SD)					
	
	Intervention			Control		
	
	Baseline	3 months	Difference	Baseline	3 months	Difference
Total Stress Score	12.33 (5.37)	14.33 (5.87)	2.00 (3.51)	12.50 (3.18)	15.63 (3.54)	3.13 (2.70)*

Total Decisional Conflict Score	34.22 (16.08)	29.22 (15.02)	-5.00 (16.42)	31.04 (18.73)	28.96 (18.59)	-2.08 (15.0)‡

Knowledge Scores	6.47 (1.41)	7.07 (1.71)	.60 (1.76)	6.63 (1.45)	6.88 (1.15)	.25 (1.06)≠

*P = 0.23 (*t*-test based on logarithms of right-skewed data)

‡ *P *= 0.23 (*t*-test based on logarithms of right-skewed data)

≠ *P *= 0.17 (*t*-test based on ranks of left-skewed data)

**Table 3:** Intervention results - per protocol analysis results (n = 29, excepting DCS n = 26)

	Mean (SD)					
	
	Intervention			Control		
	
	Baseline	3 months	Difference	Baseline	3 months	Difference
Total Stress Score	12.33 (5.37)	14.08 (5.87)	-2.38 (3.57)	12.50 (3.18)	15.63 (3.54)	3.13 (2.70) *

Total Decisional Conflict Score	34.22 (16.08)	28.21 (14.88)	-6.01 (17.40)	31.04 (18.73)	29.49 (20.39)	-1.55(15.70)‡

Knowledge Scores	6.54 (1.45)	7.15 (1.82)	-.62 (1.8)	6.63 (1.45)	6.88 (1.15)	-.25 (1.06) ≠

*P = 0.31 (*t*-test based on logarithms of right-skewed data)

‡ *P *= 0.29 (*t*-test based on logarithms of right-skewed data)

≠ *P *= 0.15 based on ranks of left-skewed data)

## Stage 2: Results 2

The mean scores, standard deviations and *t-*test results for the three key measures are presented in Table 2. Many carers in both groups had levels of decisional conflict (score above 25) that would impede decision making. Baseline levels of decisional conflict suggest 73% (intervention group) and 80% (control group) of carers would have some decisional difficulty and delay in making community service decisions. There were no statistically significant changes between groups' respite option preferences or their decision control preferences, though both groups moved away from Day Care Respite towards Short-Term Respite, and away from making solo decisions towards shared decision making.

Between-group change scores were used to evaluate decision aid effects owing to the small sample size. Between-group changes scores provide an indication of change by comparing the change exhibited by carers receiving the decision aid, with change exhibited by those receiving usual care.

Overall the findings indicate this low-cost intervention has the potential to play a role in reducing burden and decisional conflict among dementia carers. Table 2 demonstrates that, using the mean change between baseline and three month follow-up, the intervention group had less increase in burden, a greater decrease in decisional conflict and a greater increase in knowledge compared with control group participants. Table 3 shows that a per protocol analysis (with data missing for 2 intervention participants and 3 control participants for DCS only) retains the pattern of changes for intervention participants.

Considering the individual decisional conflict subscales, with the exception of one subscale, the baseline means are greater than 25, the level that is known to delay decision making (see Table 4). Participants of both groups felt more informed, better supported, less uncertain and that they made more effective decisions. Carers who used the DA gained a greater increase in value clarity (mean change -.83) compared with the decrease in value clarity found in the control group after three months (mean change +2.34). Control group participants though had greater increase in support and uncertainty subscales. None of these results reached statistical significance.

**Table 4:** Decisional Conflict Subscale mean change baseline to 3 months (n = 31)

	Participant group					
	
	Intervention			Control		
	
	Baseline	3 months	Difference	Baseline	3 months	Difference
**Decisional Conflict Subscales (0-100)**						

*Values clarity *subscale						

	31.67	30.83	-.83	20.31	22.66	2.34

*Informed *subscale						

	40.56	28.89	-11.67	35.42	28.12	-6.11

*Support *subscale						

	27.22	22.22	-5.00	30.73	25.00	-5.73

*Uncertainty *subscale						

	42.22	41.11	-1.11	39.58	37.50	-2.08

*Effective decision *subscale						

	30.00	25.00	-5.00	26.95	24.61	-2.34

**Preferred respite option n (%)**						

In-home respite	7 (46.7)	7 (46.7)	0 (0.0)	6 (37.5)	7 (43.75)	-1 (6.25)

Day respite	3 (20.0)	0 (0.0)	-3 (-20.0)	4 (25.0)	1 (6.25)	-3 (18.75)

Short-term residential respite	5 (33.3)	8 (53.3)	3 (20.0)	6 (37.5)	8 (50.0)	2 (12.5)

**Decision Control Preferences n (%)**						

I make the decision	7 (46.7)	2 (15.4)	-5	5 (50.0)	3 (31.3)	-2

I make the final decision after seriously considering the opinion/s of other/s	5 (33.3)	5 (38.5)	0	8 (30.0)	8 (50.0)	0

I share responsibility for deciding	3 (20.0)	6 (46.2)	3	3 (20.0)	5 (18.8)	2

Someone else makes decision, but... consider my opinion	0 (0)	0 (0)	0	0 (0)	0 (0)	0

I leave all decisions to someone else	0 (0)	0 (0)	0	0 (0)	0(0)	0

## Discussion

Developing DAs for surrogate care decisions with many value based complexities is a relatively new use of the DA medium. This study suggests that the Gold Book DA provides a form of decision support that dementia carers do not otherwise receive. However, providing knowledge about choices can be more difficult when compared to DAs that offer relative risk or research based outcome comparative information about choices.

Our sample of carers had high levels of decisional conflict in comparison to other study participants such as those for bowel cancer screening [41], prostate specific antigen test, ischaemic heart disease and hormone therapy (as reported in [42]). This outcome highlights the potential benefit from decision support for this group.

Mitchell et al. [43] developed and tested a decision aid for surrogate decision makers around the issue of long-term feeding options in the cognitively impaired. In a discussion that accords with our study, they noted the 'time-sensitive' nature of feeding tube decisions, influenced by the possibility of sudden changes in patients' conditions and the subsequent high levels of emotional upset. Like them, we feel that the DA may have great potential as an aid that allows carers to revisit and reconsider options. A further difference in the context of the DA is that the respite care choices being made by carers of PWD are not mutually exclusive. This has synergies with Raynes-Greenow et al.'s [44] use of a DA to help women make birthing analgesia decisions. Stated preferences for respite services did change in both groups over the three month project. This development was almost certainly linked to the deteriorating trajectory of dementia, further indicating the potential of DAs in the community setting to help surrogate decision makers revise decisions.

The RCT findings must be interpreted with caution due to the small sample size and low recruitment rate. It is possible that the results are due to chance and therefore replication with larger samples is necessary. Even so, the findings trend in a direction that suggests that the DA may be beneficial for carers and their decisional ability. Compared with carers unsupported by our DA, carers who used the GOLD Book demonstrated superior changes on measures of carer burden, decisional conflict and dementia knowledge. The results did not reach statistical significance.

Based on our pilot data, we calculate that a study with 88 participants completing the trial in each arm - requiring 104 subjects in each arm to allow for 15% loss to follow-up - would provide 80% power to detect the differences observed. This pilot study highlighted the difficulty of recruiting community based carers who are frequently busy and stressed. Our recruitment strategy was most successful when carers were directly contacted by a service they used and we recommend that strong support from service organisations is essential when recruiting community based carers. Even with this strong support, we feel a larger study would need to contact 1056 carers in order to carry out a two-arm RCT assuming a participation rate of 25%.

As a result of the pilot, we would add a carer reported measure of the severity of the care-recipient's dementia, to facilitate future targeting of the DA to those who will most benefit. Further we would add a qualitative follow-up interview to allow triangulation of data results and a deeper understanding of the patterns of DA use by carers.

## Conclusions

We have identified an important decisional support intervention gap for carers of PWD making service decisions. There is evidence that these are difficult decisions for carers and that there is a need for support that can reduce carer burden and unmet need through improved decisional ability. We have developed and piloted the DA as an intervention to fill this gap and identified the need for a more robust account of the effects of this intervention to be pursued. Future research should also examine the potential economic benefits from the use of decisional support for carers of people with dementia.

## Competing interests

The authors declare that they have no competing interests.

## Authors' contributions

CS generated the DA concept and with BL (with assistance from JS and AR) carried out the qualitative development study. CS, SL, JS, SQ, and AR developed the RCT pilot study, and SL project managed the research. BL carried out the pilot data analysis. CS with assistance from SL, developed the first draft of the paper, and all other authors contributed to subsequent drafts of the paper. All authors read and approved the final manuscript.

## Pre-publication history

The pre-publication history for this paper can be accessed here:

http://www.biomedcentral.com/1472-6947/12/21/prepub
